# Instrument Playing as a Cognitive Intervention Task for Older Adults: A Systematic Review and Meta-Analysis

**DOI:** 10.3389/fpsyg.2019.00151

**Published:** 2019-02-18

**Authors:** Soo Ji Kim, Ga Eul Yoo

**Affiliations:** ^1^Music Therapy Education, Graduate School of Education, Ewha Womans University, Seoul, South Korea; ^2^Department of Music Therapy, Graduate School, Ewha Womans University, Seoul, South Korea

**Keywords:** instrument playing, older adults, cognitive aging, cognitive engagement, systematic review

## Abstract

The aim of this meta-analysis was to review studies that applied musical instrument playing as an intervention to improve cognitive functioning of older adults with and without cognitive impairment. English-language articles published between 1990 and 2018 were searched using electronic databases. Music therapy journals were also hand searched for relevant research. Inclusion criteria for participants were older adults, ages 60 years and older, and any clinical diagnosis of cognitive impairment had to be due to aging. Searches used combinations of the following keywords: older adults, instrument playing, and cognitive outcomes measures. A total of 10 studies that met the inclusion criteria were included in the final analysis: five studies with healthy older adults, two with older adults with mild cognitive impairment (MCI), two studies with older adults with dementia, and one study with both healthy older adults and older adults with MCI. The results of this meta-analysis demonstrated that different types of cognitive involvement were demanded from instrument playing. Furthermore, depending on the type of involvement, a target cognitive domain was found to be differentially impacted by the instrument playing intervention. This study supports using different types of instrument playing for interventions targeting specific cognitive domains of older adults with varying levels of cognitive aging.

## Introduction

Age-related decline results in the general slowing of mental processing and affects the ability to control and coordinate cognitive processes (Verhaeghen et al., 2003; Fernandez-Duque and Black, 2006; Boisgonitier et al., 2013). This cognitive decline is aggravated by the onset or progression of cognitive impairment, such as dementia, and is most noticeable when it impacts active control of attentional resources that significantly affect everyday life tasks, including walking (Fernandez-Duque and Black, 2006; Sheridan and Hausdorff, 2007; Foley et al., 2011). This aggravation limits independent engagement in activities in later life, which often leads to decreased quality of life and increased individual and societal costs for healthcare. Given that cognitive decline can progress to a severe level of cognitive impairment, the development of effective interventions for improving or maintaining cognitive functioning of older adults is of particular interest to researchers, practitioners, and individuals experiencing such decline and their families (Bahar-Fuchs et al., 2013).

Reviews of interventions for improving cognitive performance in older adults have found that these interventions range from cognitively stimulating activities to cognitive training (Martin et al., 2011; Kelly et al., 2014). Cognitive training directly and specifically targets cognitive tasks and has been documented to significantly improve various cognitive domains, such as memory, executive function, and attention/processing speed (Martin et al., 2011; Reijnders et al., 2013). However, transfer of intervention effects to everyday life activities has not been evidenced. The applicability of cognitive training to older adults with expression or progression of relatively severe cognitive impairment remains inconclusive. Compared to cognitive training, cognitive engagement interventions involve cognitively stimulating activities that may be a part of daily living, such as reading or music activities (Park et al., 2007; Martin et al., 2011). While active engagement in cognitively, physically, and socially stimulating activities of everyday life was found to reduce the risk of further cognitive impairment (Mangialasche et al., 2012), systematic analysis of how such approaches lead to expected outcomes has not been attempted.

Music activities and music-based interventions have great potential to be effectively applied to facilitate cognitive engagement of older adults. Active and intensive engagement in music activities was repeatedly reported to affect cognitive processing in later life, which is supported by music-induced brain plasticity (Moussard et al., 2016). For older adults, maintained engagement in music activities is also predictive of their verbal and visuospatial processing (Hanna-Palddy and Gajewski, 2012). In terms of the potential for stimulation, music activities affect the cognitive, social, emotional, and physical domains throughout the lifespan and are easily applied to activities of everyday life (Ueda et al., 2013). As such, engagement in music activities was found to immediately stimulate cognitive performance, such as memory (Han, 2016) and reduce attentional load from intensive exercises leading to increased engagement in target activities (Kim, 2017).

Instrument playing involves motor and cognitive functions simultaneously. Instrument playing requires unimanual movement, bimanual movement, finger movement, or whole upperlimb motor movement to handle instruments (e.g., striking a drum, pressing the keys of piano, or shaking small instruments). Also, additional tasks may be integrated (e.g., remembering the rhythm to be played or learning a new way of playing the instrument). Given the increased emphasis on the interplay between cognitive and motor functioning in cognitive control in older adults (Fernandez-Duque and Black, 2006; Al-Yaha et al., 2011; Agmon et al., 2014), instrument playing involving both functions could be an effective agent for cognitive intervention to target older adults. For example, the concurrent processing of different combinations of musical elements and motor movements while switching between tasks may facilitate cognitive flexibility (Moradzadeh et al., 2015). It has been reported that engagement in intensive group instrument playing, such as in an orchestra, might address decline in inhibitory control of cognitive processing in advanced age (Vromans and Postma-Nilsenová, 2016).

Despite the potential of instrument playing for cognitive intervention, its easy accessibility to older populations, and its low demand on mental resources (Solé et al., 2014), there has not been a systematic analysis of the various types of playing tasks applied to this population. Different types and levels of tasks are presumably associated with different levels of cognitive aging and target cognitive outcomes (Li et al., 2016). Given the broad range of possible instrument playing tasks, there are calls for the systematic analysis of the factors involved, particularly as related to instrument playing as cognitive engagement for older adults. Investigation into which factors influence outcomes and how a specific aspect of instrument playing can be selected and constructed for targeted goal would delineate the implications for the specific design and implementation of an intervention based on the target individual's level of cognitive aging and unique needs.

Therefore, this study aimed to systematically review the research on instrument playing with older adults with and without cognitive impairment. The focus of this study was on analyzing the research in terms of the type of cognitive stimulation or cognitive engagement included in the instrument playing tasks and the differential effects associated with the type of cognitive domain targeted.

## Methods

### Search Strategy

English-language articles published between 1990 and 2018 were searched from January through August 2018 using electronic databases, including CINAHL, Cochrane Central Register of Controlled Trials (CENTRAL), and PubMed. Keywords used in the electronic search included older adults, elderly, seniors, aging, cognitive aging, cognitive impairment, dementia, Alzheimer's disease, mild cognitive impairment (MCI) for population-specific terms; music, music therapy, instrumental playing, musical instrument, keyboard, piano, drumming, and percussion for intervention-specific terms; and cognitive, cognition, memory, working memory, recall, executive function, visuosptial perception, verbal fluency, attention, attentional control, and processing speed for outcome-specific terms. Music therapy journals were also hand searched for relevant research published during the same time period. After initial review of titles and abstracts, duplicate studies and obviously irrelevant studies were excluded. For potentially relevant articles, full texts were retrieved and examined to ensure they met the inclusion criteria.

### Inclusion Criteria

Inclusion criteria for participants were older adults, ages 60 years or older. While participants with a clinical diagnosis of cognitive impairment due to aging (i.e., dementia) were also included, individuals with comorbidity, such as other neurological or degenerative disorders, were excluded. Therefore, healthy older adults and older adults with MCI or dementia were included. With regard to type of intervention, studies were included if their intervention utilized instrument playing as a primary intervention method. Given that music intervention could involve multiple musical behaviors, such as listening and playing or singing and playing, the inclusion of another type of musical behavior did not disqualify studies. If the instrument playing was separately applied and described as a primary independent task in the intervention, the study was included. However, studies were excluded in which instrument playing was described as an auxiliary task (e.g., playing instruments while singing) or the primary task could not be identified (e.g., when the task was described as a general music activity, including both singing and instrument playing). Furthermore, in this review, studies on the effects of instrument playing compared to traditional interventions or other controlled interventions were included. To analyze the effects of instrument playing on cognitive aging, cognitive outcome measures were included, such as memory, working memory, recall, executive function, visuospatial perception, verbal fluency, attention, attentional control, and processing speed. Study designs with controlled comparison groups were eligible for inclusion in this review. Studies with randomized controlled trials, controlled clinical trials, or inclusion of a control group were included in the final analysis.

### Assessment of Risk of Bias in Included Studies

Each of the included studies was assessed for its risk of bias in terms of four items: (a) random sequence generation, (b) allocation concealment, (c) blinding of outcome assessment, and (d) incomplete outcome data addressed, depending on (a) whether all participants had an equal chance of being assigned to one of the conditions, (b) whether adequate procedures to conceal group assignment were clearly reported, (c) whether outcome assessors were blinded to the information of which intervention a participant received, and (d) whether there was a report on any participants who dropped out during the study and the reason for incomplete data. The criteria used for judging each item were based on those provided in the *Cochrane Handbook for Systematic Reviews of Interventions* (Higgins and Green, 2011).

### Statistical Analysis

Review Manager (RevMan V.5.3) was used for conducting the statistical analyses. For each outcome variable in each included study, standardized mean differences (SMD) were calculated with 95% confidence intervals. For measurements that evaluated the time to complete a test and indicated more positive effects with smaller values, the mean values were multiplied by −1 to ensure that all the cases pointed in the same direction. For each of the cognitive outcome areas, the pooled estimate was analyzed using a fixed-effects model. An effect size of 0.2–0.4 was considered a low effect size, with 0.5–0.7 a moderate effect size, and >0.8 a large effect size (Cohen, 1992). The extent of heterogeneity across studies was determined by calculating the *I*^2^ statistic. For the statistic calculating the percentage of variability in estimated effect sizes, the extent of heterogeneity was interpreted as low, medium, or high at 25, 50, and 75%, respectively. To examine if the effect of instrument playing varied depending on the type of cognitive engagement, differences between effect sizes for each level of the variables were tested using Fisher's *Z*-test. *I*^2^ statistics were conducted to measure the extent of heterogeneity within each subgroup.

## Results

**Figure 1:**
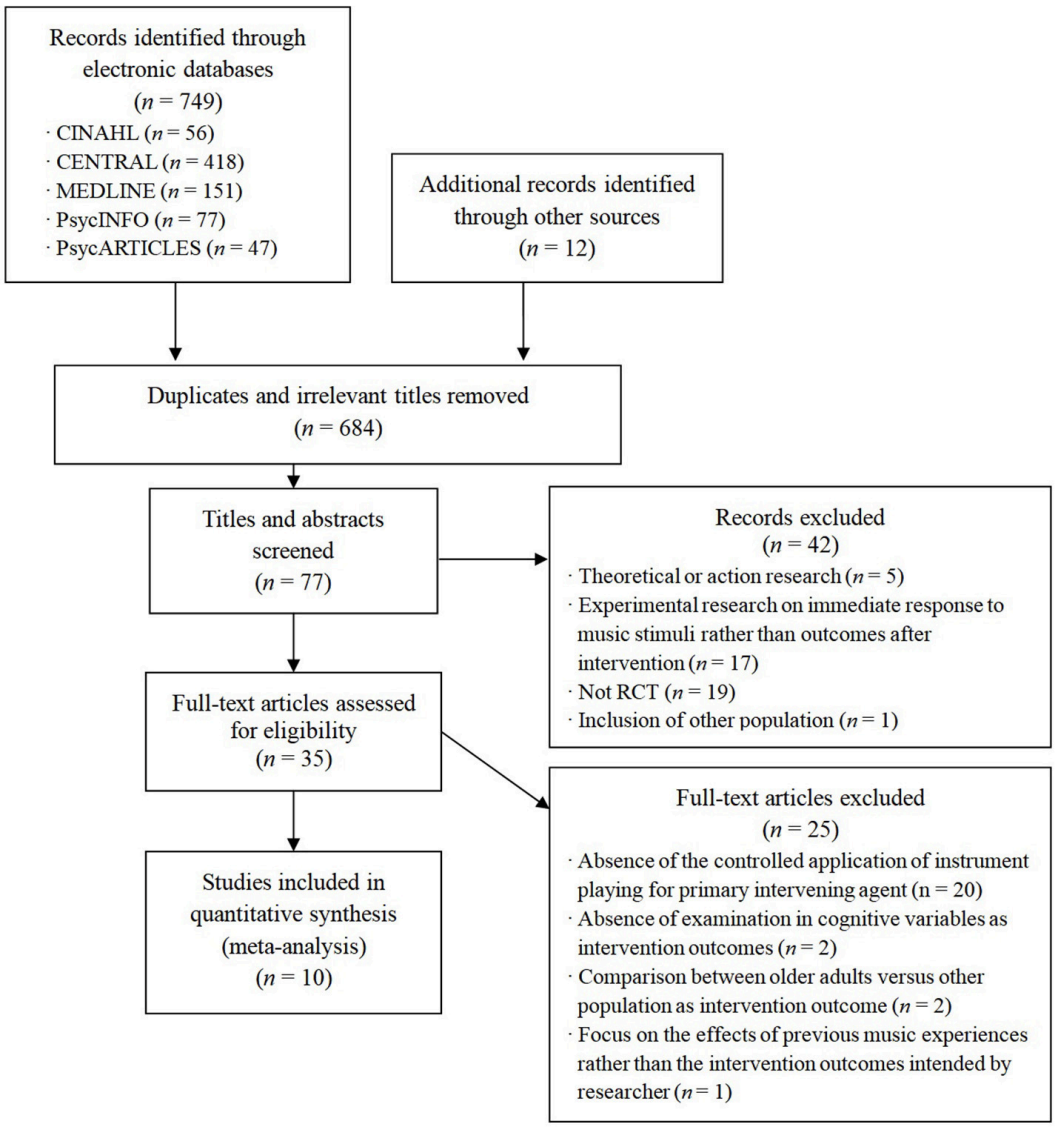
An illustration of the number of studies identified and qualified for final analysis.

A total of 761 articles were identified after initial database and hand searches. Duplicates and irrelevant studies were excluded, and 77 studies were retrieved for screening. Finally, 10 studies with a total of 635 participants were selected as meeting the inclusion criteria (see Figure 1). Five studies examined the effects of music intervention involving instrument playing on healthy older adults, two studies involved older adults with MCI, two studies targeted older adults with dementia, and one study targeted both healthy older adults and older adults with MCI. More detailed descriptive characteristics of each study are displayed in Table 1. In terms of the quality of the included studies, seven studies (70%) randomly assigned participants either to the intervention group that included instrument playing or the control group. Four studies reported that they appropriately concealed group allocation, and five studies stated that the assessors were blind to the group allocation. Meanwhile, 90% of the included studies reported how many data were missing and how they dealt with such missing data (see Table 2).

**Table 1:** Specified characteristics of included studies.

**References**	**Participant**				**Intervention**		
	**Research design**	**Level of cognitive aging**	***N***	**Mean age**	**Type of intervention**		**Intensity minutes, frequency, duration**
			**IP/Con**	**IP/Con**	**IP**	**Con**	
Biasutti and Mangiacotti, 2018	RCT	H, MCI	18/17	83.39/83.76	Cognitive music training	Gymnastic activity	70 min, biweekly, 24 weeks
Bugos, 2010	Control	H	24/22	69.3/67.7	Group piano playing	Music listening	45 min, 1/week, 16 weeks
Bugos et al., 2007	RCT	H	16/15	71.4/69.6	Individual piano playing	No int.	30 min, 1/week, 6 weeks
Chen and Pei, 2018	RCT	D	15/13	77.3/77.3	Musical dual-task training	Non-musical cognitive tasks and walking exercises	60 min, 1/week, 8 weeks
Chu et al., 2014	RCT	D	49/51	82	Group MT	Usual care	30 min, 2/week, 6 weeks
Doi et al., 2017	RCT	MCI	67/67	76.2/76.0	Cognitive music activity	Health education	60 min, 1/week, 40 weeks
Hars et al., 2014a	RCT	H	23/29	76/73.5	Multitask training	Delayed int.	60 min, 1/week, 45 weeks/year, 4 years
Hars et al., 2014b	RCT	H	66/68	75/76	Multitask training	Delayed int.	60 min, 1/week, 25 weeks
Seinfeld et al., 2013	CCT	H	13/16	69.3/69.6	Group piano playing	Leisure activity	90 min, 1/week, 4 months
Shimizu et al., 2017	RCT	MCI	34/10	74.9/73.3	Movement MT	Single-training task (exercise)	60 min, 1/week, 12 weeks

*IP, instrument playing; Con, control group; RCT, randomized controlled trials; H, healthy older adults; MCI, mild cognitive impairment; D, dementia; Int, intervention; MT, music therapy*.

**Table 2:** Quality assessment of included studies.

**References**	**Random allocation**	**Allocation concealment**	**Blinding of outcome assessors**	**Incomplete outcome data**
Biasutti and Mangiacotti, 2018	Y	N	N	Y
Bugos, 2010	N	N	N	N
Bugos et al., 2007	Y	N	N	Y
Chen and Pei, 2018	Y	N	Y	Y
Chu et al., 2014	Y	Y	N	Y
Doi et al., 2017	Y	Y	Y	Y
Hars et al., 2014a	Y	Y	Y	Y
Hars et al., 2014b	Y	Y	Y	Y
Seinfeld et al., 2013	N	N	N	Y
Shimizu et al., 2017	N	N	Y	Y

*Y, Yes; N, No*.

The instrument playing task in each study was analyzed in terms of the type of instrument playing and the subcomponents of the instrument playing task (see Table 3). The type of instrument playing included the following: instrumental improvisation, piano instruction, percussion while memorizing rhythms or reading music scores, group instrument playing in a specified rhythm or at specified timing, and instrument playing while walking. Among the included studies, five studies included the instrument playing task exclusively, while the other five studies involved instrument playing in parallel with another task. All five studies involving another music task used instrument playing and singing tasks independently but gave them equal importance. In analyzing the subcomponents of the instrument playing tasks in the included studies, instrumental improvisation included a cognitive component in creating rhythms. Playing only to a specified timing or reading specifically designed music scores (e.g., color-coded scores) were required in other types of rhythm playing or in playing percussion. Piano playing tasks involved memorizing rhythms, reading musical notation, and learning music theory. Multitask training consisted of concurrent motor (i.e., walking or specified body movements) and cognitive components (i.e., changing movement in response to changes in music or playing the held instrument only to the specified timing).

**Table 3:** Analysis of the components of cognitive stimulation.

**References**	**Type of application of IP tasks**	**Type of parallel music task**	**Type of IP task**	**Instruments**	**Subcomponents of IP tasks**		
					**Motor**	**Motor**	**Cognitive**
Biasutti and Mangiacotti, 2018	P	S	Instrumental improvisation	Percussion instruments (NS)	Handling instruments	–	Creating rhythms
Bugos, 2010	E	NA	Group piano playing	Piano	Finger key-pressing	–	Reading musical notation and learning music theories
Bugos et al., 2007	E	NA	Individualized piano playing	Piano	Finger key-pressing	–	Reading musical notation and learning music theories
Chen and Pei, 2018	P	S	Musical dual-task training	Percussion instruments	Handling instrument	Walking	Maintaining a steady beat and playing only for a certain section of a song
Chu et al., 2014	P	S	Rhythm playing	Triangles, clappers, maracas, handbells, tambourines, and color sound bell	Handling instruments	–	Recognizing different types of music and playing in a specified way (rhythm; timing)
Doi et al., 2017	E	NA	Playing percussions	Percussion (e.g., conga)	Handling instruments	–	Memorizing rhythms and reading music scores
Hars et al., 2014a	P	S	Multitask training	Percussion (N.S)	Handling instruments	Walking	Changing movement in response to changes in music and performing the concurrent tasks while walking
Hars et al., 2014b	P	S	Multitask training	NR	Handling instruments	Walking	Changing movement in response to changes in music and performing the concurrent tasks while walking
Seinfeld et al., 2013	E	NA	Group piano playing	Piano	Finger key-pressing	–	Reading musical notation and learning music theories
Shimizu et al., 2017	E	NA	Multitask movement MT	Naruko (clappers)	Handling instruments	Specified body movement	Imitating movements

*IP, instrument playing; E, exclusive; P, parallel (indicates that the study included another music task in parallel with instrument playing); NA, not applicable; MT, music therapy; NS, not specified; NR, not reported*.

The included studies involved playing tasks that were categorized as either immediate engagement or sustained engagement. Given that all of the tasks required novel and challenging activities and accordingly demanded the processing of new knowledge (rather than involving familiar tasks that could be performed based on existing knowledge), they were classified as productive engagement (Park et al., 2007). Meanwhile, depending on the period of time necessary to accomplish the instrument playing task, the tasks were subcategorized into immediate (e.g., instrument improvisation and multitasking) vs. sustained engagement (e.g., piano instruction). Furthermore, the playing tasks categorized as immediate engagement were sub-classified into tasks with additional cognitive (e.g., creating rhythms or reading color-coded scores that indicated the timing of playing) vs. additional motor tasks (e.g., walking). A summary of each category is displayed in Table 4.

**Table 4:** Example of the category of cognitive involvement.

**Type**	**Example of instrument playing**	
Immediate engagement with the addition of cognitive tasks	Striking or shaking instruments while creating a new rhythm or while following the timing indicated by color-coded scores	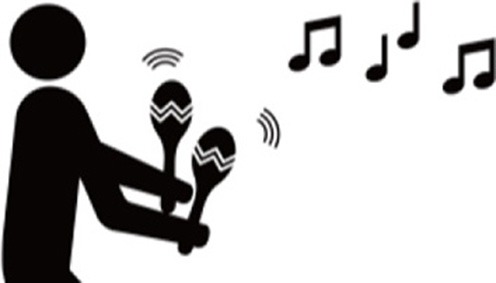
Immediate engagement with the addition of motor tasks	Striking instruments while walking and changing speed of movement in response to changes in music	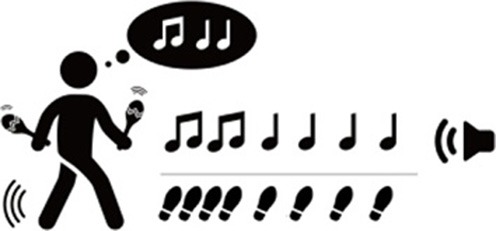
Sustained engagement	After learning music theory and score reading, playing the piano while reading a score based on memorized information	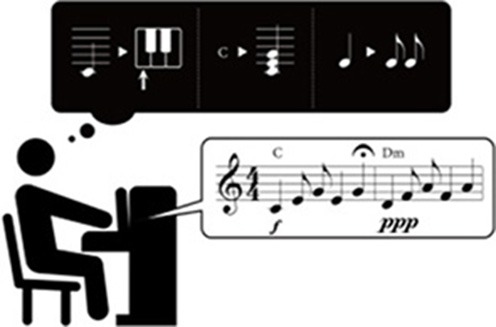

In terms of target population, different types of cognitive involvement were applied (Table 5). For healthy older adults, sustained engagement was included in three studies and immediate engagement was included in three studies (one with additional cognitive tasks and two with additional motor tasks). All of the studies with participants with MCI and dementia included immediate engagement. For the two studies on older adults with dementia, one included an additional cognitive task and the other an additional motor task. For the three studies on older adults with MCI, two included cognitive tasks along with instrument playing and one included motor tasks.

**Table 5:** Type of cognitive involvement in the included studies depending on target population.

**Target population**	**Type of cognitive involvement (number of studies)**	**References**
Healthy older adults	Immediate/cognitive (1)	Biasutti and Mangiacotti, 2018
	Immediate/motor tasks (2)	Hars et al., 2014a,b
	Sustained (3)	Bugos et al., 2007; Bugos, 2010; Seinfeld et al., 2013
Older adults with dementia	Immediate/cognitive (1)	Chu et al., 2014
	Immediate/motor tasks (1)	Chen and Pei, 2018
Older adults with MCI	Immediate/cognitive (2)	Doi et al., 2017; Biasutti and Mangiacotti, 2018
	Immediate/motor tasks (1)	Shimizu et al., 2017

*Immediate/cognitive, immediate engagement with the addition of cognitive tasks; immediate/motor, immediate engagement with the addition of motor tasks; sustained, sustained engagement; MCI, mild cognitive impairment*.

The measurements used in the included studies in relation to the targeted cognitive domain are displayed in Table 6. When the target cognitive domain was categorized into general cognition, processing speed, memory, attentional control, verbal fluency, executive function, or visuospatial perception, the studies targeting healthy older adults and older adults with MCI showed similar trends in targeted subdomains and types of measurement. Meanwhile, for studies with older adults with dementia, only general cognition and attentional control were targeted. The measurements in these studies included the Mini-Mental State Examination (MMSE) and Trail Making Test-A (TMT-A), which may indicate the applicability of such measurements for individuals with relatively severe cognitive impairment.

**Table 6:** Target cognitive domain and measurement used in the included studies.

**Cognitive domain**	**Measures used for each target population**		
	**Healthy older adults**	**MCI**	**Dementia**
General cognition	MMSE	MMSE	MMSE
Processing speed	AMT; PASAT	AMT	–
Memory	DSF; DSB; Letter number; SSF; SSB	Story memory; word memory	–
Verbal fluency	D-KEFS Verbal fluency; VFL	VFL	–
Attentional control	TMT-A	TMT-A	TMT-A
Executive function	FAB; Stroop; TMT-B	TMT-B	–
Visuospatial	Block design; CDT; SDMT; TMT-A	CDT	–

*MMSE, Mini-Mental State Examination; AMT, Attentional Matrices Test; PASAT, Paced Serial Addition Task; DSF, Digit Span Forward; DSB, Digit Span Backward; SSF, Spatial Span Forward; SSB, Spatial Span Backward; D-KEFS, Delis-Kaplan Executive Function System; VFL, Verbal Fluency-Letter; TMT, Trail Making Test; FAB, Frontal Assessment Battery Test; CDT, Clock Drawing Test; SDMT, Symbol Digit Modalities Test*.

The present analysis also investigated whether there were differences in outcomes for the different types of instrument playing tasks. For overall effects on each of seven cognitive domains, low effect sizes were obtained for general cognition (*d* = 0.28), memory (*d* = 0.26), verbal fluency (*d* = 0.19), attentional control (*d* = 0.22), executive function (*d* = 0.25), and visuospatial perception (*d* = 0.19). A large effect size was observed for processing speed (*d* = 0.94). Substantial heterogeneity was found with processing speed, memory, and executive function (61–84%), while low heterogeneity was found with general cognition, verbal fluency, attentional control, and visuospatial perception.

**Figure 2:**
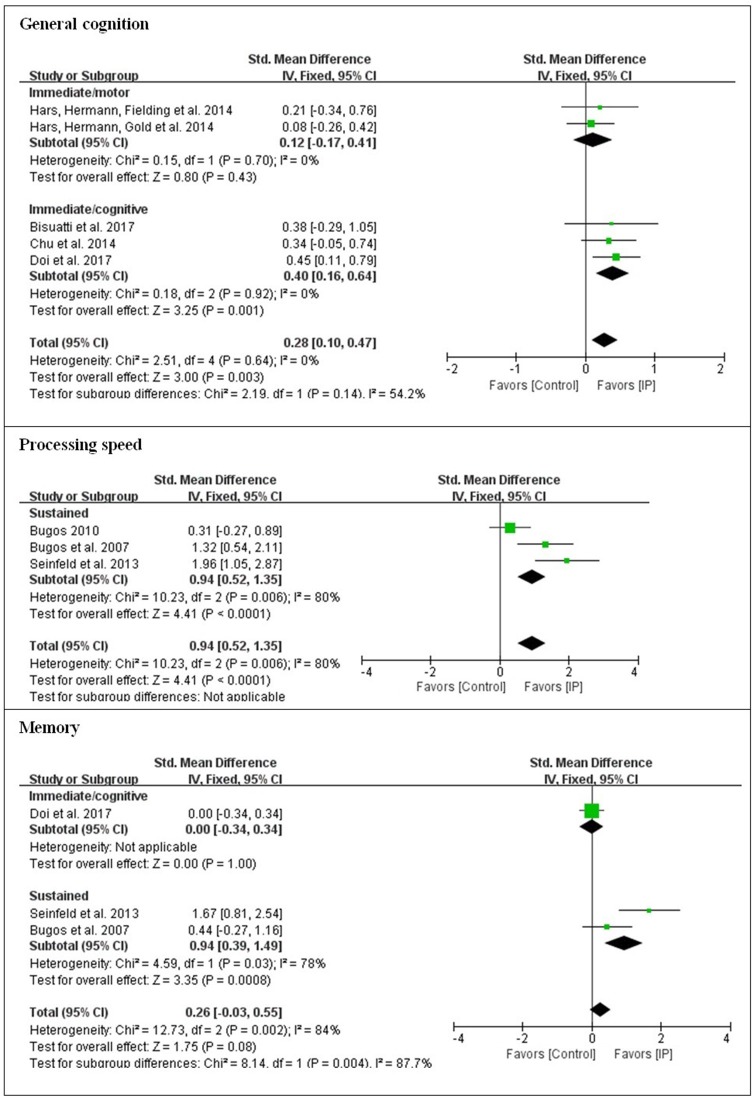
Effect sizes of instrument playing in the domain of general cognition, processing speed, and memory depending on the type of cognitive engagement.

**Figure 3:**
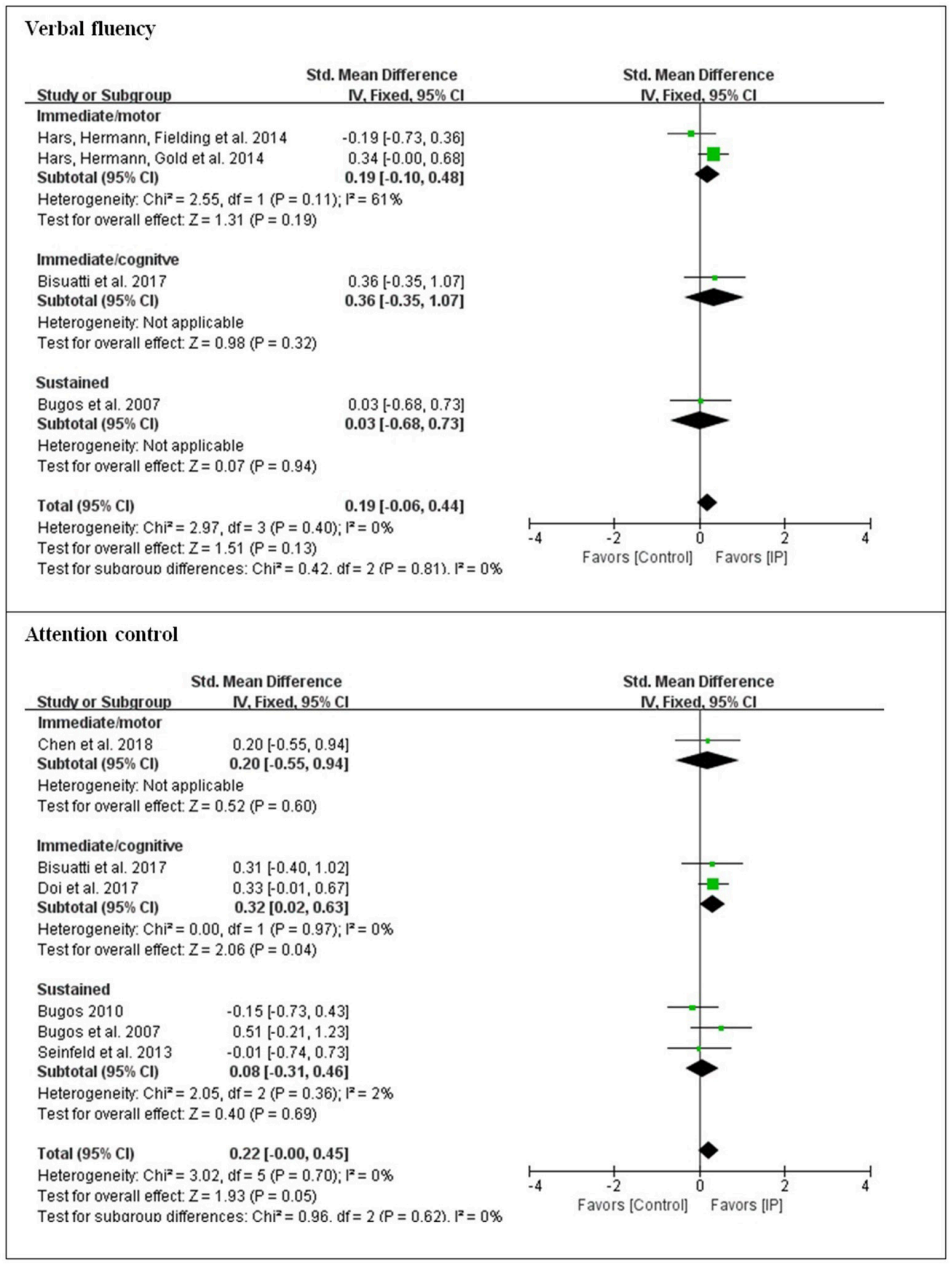
Effect sizes of instrument playing in the domain of verbal fluency and attentional control depending on the type of cognitive engagement.

**Figure 4:**
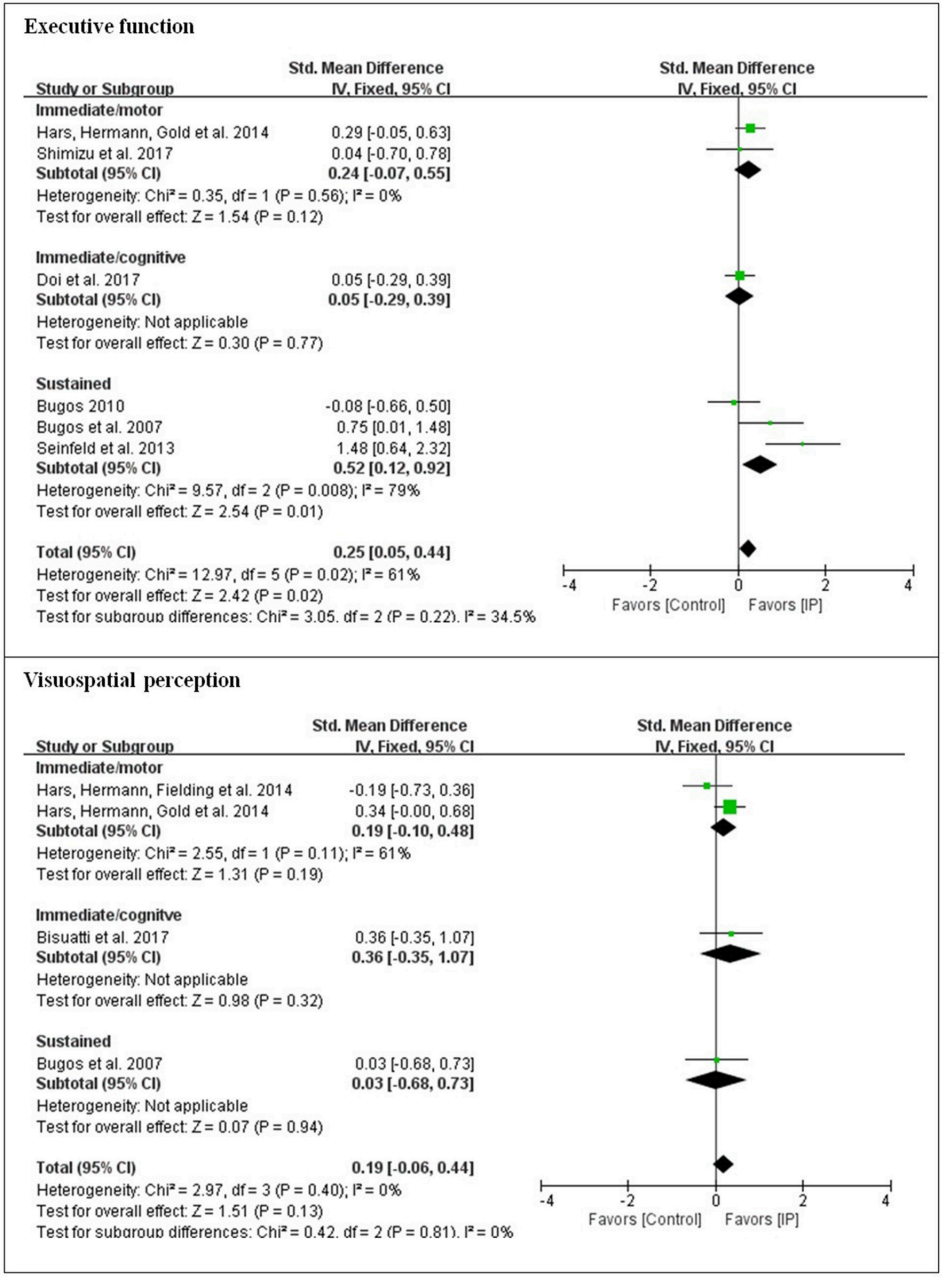
Effect sizes of instrument playing in the domain of executive function and visuospatial perception depending on the type of cognitive engagement.

In order to see which type of cognitive domain might be addressed more effectively with which type of cognitive involvement, subgroup analysis was conducted with the moderating variable of the type of cognitive involvement [i.e., immediate engagement with the addition of cognitive tasks (immediate/cognitive), immediate engagement with the addition of motor tasks (immediate/motor), and sustained engagement (sustained)] in each cognitive domain. For general cognition, although immediate/cognitive engagement elicited a greater effect size than immediate/motor engagement, it was at a low level (*d* < 0.5). For processing speed, only instrument playing as sustained engagement was applied, and the effect size was large. For memory, sustained engagement was observed with a large effect size (*d* = 0.94), which was greater than immediate/cognitive engagement. The domains to which all three types of cognitive involvement were applied were verbal fluency, attentional control, executive function, and visuospatial perception. Except for executive function, the domains that targeted immediate/cognitive engagement led to greater effect sizes than the other two types of cognitive involvement, but the effect sizes were small (*d* < 0.5). For executive function, sustained engagement was measured with a moderate effect size (*d* = 0.52), which was greater than with the other two types of cognitive involvement (see Figures 2–4).

Finally, in terms of maintenance of intervention effects, this study analyzed how intervention outcomes were maintained after the intervention was terminated (see Table 7). Only three of 10 studies obtained follow-up data. The study by Bugos et al. (2007) demonstrated that processing speed/attention measured by the Digit Symbol Test and executive control measured by the Trail Making Test-B were enhanced at follow-up as compared to posttest, and such changes were significantly different from the no intervention group that showed a decline in such measures. However, another study (Chu et al., 2014) did not support maintenance of the intervention effects. The other study (Hars et al., 2014a) did not conduct a statistical analysis on the follow-up data for the cognitive domain, and only performed such an analysis on motor domain measures.

**Table 7:** Analysis of maintenance of intervention effects in the included studies.

**References**	**Time point of follow-up test**	**Measured cognitive domain**	**Measurement**	**Result**
Chu et al., 2014	One month after termination	General cognition	MMSE	While Ex showed no significant decline in MMSE from post-intervention to follow-up, no report on the statistical analysis for Con
Hars et al., 2014a	Three years after termination	General cognition Visuospatial perception	MMSE CDT	No statistical analysis on the cognitive measures and group comparison was conducted Ex and Con showed similar results such as slight decrease in MMSE and slight increase in CDT at follow-up compared to post-intervention
Bugos et al., 2007	Three months after termination	Processing speed/attention Executive function	Digit symbol TMT-B	Improvement in digit symbol and TMT-B from post-intervention to follow-up was observed in Ex, and such changes were significantly different from the Con

*Ex, Experimental; Con, Control; MMSE, Mini-Mental State Examination; CDT, Clock Drawing Test; TMT, Trail Making Test*.

## Discussion

This study aimed to systematically review the literature on musical instrument playing for improving cognitive performance in older adults with cognitive aging. A total of 10 studies with healthy older adults, older adults with MCI, and older adults with dementia met the inclusion criteria and were included in the final analysis. The results of the current study highlight how instrument playing was applied as a cognitive engagement for enhancing or maintaining cognitive involvement in older adults with different levels of cognitive functioning.

First, instrument playing was found to involve cognitive components along with motor components. Although instrument playing basically involves motor tasks (e.g., handling objects or hand/finger movements), the instrument playing tasks in the included studies demanded additional cognitive tasks by requiring older adults to operate instruments in a specified way. Such components included sustaining attention toward external stimuli, recognizing changes in the stimuli, and selecting the expected actions in response to such input. These results indicate that instrument playing has great potential for active engagement with older adults. Compared to receptive engagement based on existing knowledge or familiar activation, productive engagement involves novel information and challenging tasks (Park et al., 2007). Given that productive engagement facilitates the cognitive process, more particularly cognitive control and executive processes, instrument playing as productive engagement may contribute to developing and expanding the resources for cognitively stimulating older adults with cognitive aging.

Furthermore, instrument playing in the literature was found to take the form of immediate engagement or sustained engagement. When immediate cognitive engagement was required from instrument playing, the participants were asked to take part in additional cognitive or motor tasks. It is noteworthy that the cognitive involvement in instrument playing differed depending on the level of cognitive aging of the participants. While instrument playing was applied to demand both immediate and sustained engagement from healthy older adults, no studies were found that required new learning over a certain period of time for older adults with cognitive impairment (i.e., MCI or dementia). Although there were not an adequate number of studies across participants with different levels of cognitive aging to reach firm conclusions, instrument playing was constructed to require a relatively limited range of cognitive involvement in older adults who experienced the onset or progression of cognitive impairment. In terms of target cognitive domain and outcome measurements, only general cognition and attentional control were targeted with older adults with dementia, and the measurements used only included the MMSE and TMT-A. This might be attributed to the fact that cognitive measurements of individuals with mild-to-moderate cognitive impairment have limitations.

Still, this result underscores the need for further application of instrument playing as a medium for cognitive stimulation specific to cognitive decline. It is worth noting that cognitive rehabilitation has become a critical area of focus of intervention with older adults even with moderate-to-severe dementia (Woods et al., 2012; Bahar-Fuchs et al., 2013). Moreover, the population with dementia can benefit from training that targets more complex cognitive functioning, including dual task performance as executive control of cognitive and motor tasks (Schwenk et al., 2010). Given that instrument playing can be applied with a modifiable level of cognitive involvement, more diversified application of instrument playing is needed.

In this study, each type of instrument playing was found to address different cognitive domains by requiring varying levels of cognitive involvement. After intervention requiring a new task (i.e., instrument playing) enhanced processing speed, memory, and executive function were evidenced. This supports that such a task might tap into the ability to integrate new information into the existing cognitive structure, access such information, and retrieve it from a preserved source (Hertzog et al., 2003). Repetitive utilization of cognitive resources to acquire novel information (e.g., how to read a music score to play the piano) and application of that information through task performance with progressively increased complexity (Bugos et al., 2007; Bugos, 2010) can effectively intervene in improving or maintaining those cognitive abilities. Furthermore, instrument playing that combines cognitive and motor aspects and facilitates intrinsic motivation to search for resources to accomplish novel and challenging tasks can impact the ability to regulate thoughts or behaviors while continuously tracking external stimuli and information. This result indicates that instrument playing requiring sustained engagement for new learning may effectively intervene in cognitive flexibility and executive control.

Meanwhile, instrument playing as immediate engagement along with additional cognitive tasks was found to affect general cognition, verbal fluency, attentional control, and visuospatial perception more than other types of cognitive involvement, but such effects were not at a substantial level. This indicates that this approach could stimulate cognitive performance in older adults in general, but it might not be sufficient to be transferred to other cognitive domains or performance of daily living activities. Instrument playing as immediate engagement with addition of motor tasks was not found to have large effect sizes. This might be attributed in part to the fact that the included studies measured changes in cognitive control via motor-related parameters (e.g., changes in temporal parameters of walking while walking and performing cognitive tasks concurrently). The measurements included in such studies might not directly represent changes in the involved cognitive functioning. As such, the results of this meta-analysis should be generalized with caution, since this study did not directly compare the effects of different types of instrument playing. How instrument playing can be differently constructed depending on the severity of cognitive impairment and disease progression and which outcomes its application can bring forth will need to be systematically investigated.

Finally, the results of this current review support that transfer of intervention effects to other cognitive domains is promising. As such, instrument playing has the potential to transfer to everyday life activities by cognitively stimulating and affecting lifestyle factors in older populations in their natural environment, compared to cognitive training that targets specified sets of cognitive tasks. However, follow-up data were available in only four of the 10 studies. Although a few studies demonstrated that the gained outcomes could be maintained after the intervention ended, there were not consistent and conclusive results across the studies. Accordingly, there was insufficient data to investigate the transfer and maintenance of obtained outcomes at follow-up. Further clinical research will need to address this issue by including such measures. This study supports the potential of instrument playing as a cognitively stimulating task to address the cognitive needs of older populations. However, additional studies need to be conducted that include research published in languages other than English and broader range of publications, given that intervention for aging population is of common interest worldwide and the systematic analysis of the impact of such intervention on cognitive functioning could present clinical implications.

## Conclusion

The current study proposes how instrument playing could be constructed as a cognitively stimulating task and what could be differentially expected from a specific type of instrument playing. The results suggest that instrument playing can be differentially constructed to address the diverse needs of older adults with cognitive aging. Also, this indicates that considering the level of cognitive demand is critical for the expected intervention effects. Depending on the kinds of tasks to be targeted, clinicians may be able to design diverse levels of cognitive stimulation by determining subcomponents of the tasks.

## Author Contributions

SK: research idea, research design, data analysis, and writing; GY: data collection and analysis and writing.

### Conflict of Interest Statement

The authors declare that the research was conducted in the absence of any commercial or financial relationships that could be construed as a potential conflict of interest.
